# Effect of skull shape approximations in Gamma Knife dose calculations

**DOI:** 10.1120/jacmp.v8i3.2377

**Published:** 2007-07-17

**Authors:** Anita Berndt, James Beck

**Affiliations:** ^1^ Division of Medical Physics CancerCare Manitoba; ^2^ Winnipeg Centre for Gamma Knife Surgery, Health Sciences Centre Winnipeg Regional Health Authority; ^3^ Section of Neurosurgery University of Manitoba Winnipeg Manitoba Canada; ^4^ Department of Surgery and Departments of Radiology and Physics and Astronomy University of Manitoba Winnipeg Manitoba Canada

**Keywords:** Gamma Knife skull measurements, patient contour extraction, dose calculation approximations

## Abstract

Doses in Leksell GammaPlan (Elekta, Stockholm, Sweden) are calculated for each shot by summing the contribution from the 201 radiation beams emitted by the Gamma Knife (Elekta), weighted for attenuation in the tissue traversed. The patient's head is modeled based on 24 skull measurements, from which the depth to the calculation point is determined for each beam. The limited number of measurement points associated with this approach can result in substantial discrepancies between the skull model used for dose calculations and the actual skull contour from computed tomography or magnetic resonance data. A review of 24 patients found that differences between the actual and approximated skull shape gave treatment time errors as large as 4.1%, although in most instances, the errors were less than 1%. The conclusion was that the Leksell GammaPlan head model provides a quick and convenient approach for specifying the shape of the patient's head in all but extreme cases, where discrepancies as large as ~5% can result.

PACS number: 87.53.Ly

## I. INTRODUCTION

The Leksell Gamma Knife (Elekta, Stockholm, Sweden) provides a minimally invasive treatment option for patients with brain lesions and other conditions such as arteriovenous malformations and trigeminal neuralgia. The Gamma Knife uses 201 C60o sources with a common convergence point to generate an approximately spherical dose distribution.^(1)^ Multiple isocenters (“shots”) are combined to deliver a cytotoxic dose to the lesion with high conformity and minimal radiation to the surrounding brain.

At the beginning of each treatment, a rigid frame attached to the patient's skull establishes a coordinate system that relates the treatment planning images to the Gamma Knife patient positioning system and that immobilizes the patient during the treatment. A plan is generated by adding radiation shots to cover the lesion, as visualized in the treatment planning images. Customized conformal plans can be created by

- adjusting the shot weights;
- changing the shot radius by choosing one of the four available collimator helmets (4‐, 8‐, 14‐, 18‐mm single‐beam full width at half maximum); and
- blocking selected beam channels with solid plugs to help spare critical structures.

**Figure 1:**
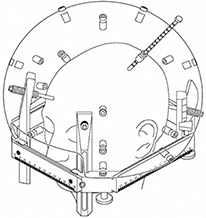
Sketch of the skull measurement procedure used to model the patient's head. The head frame, skull measurement sphere, and ruler (top left side of patient) are shown. (Image courtesy of Elekta, Inc.)

**Figure 2:**
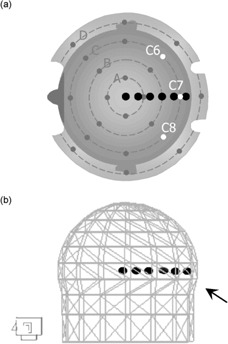
Series of single‐shot treatments (x=100 mm; y=30, 40, 50, …, 100 mm; z=100 mm) used to investigate the effect of shot position on skull model discrepancies, of which six shots are illustrated. (a) Top view of the skull measurement tool, showing the 24 measurement points (dark grey circles; white circles are measurement points C6, C7, and C8). (b) Skull model with all measurement points set to 80 mm. The arrow indicates the region in which the skull shape is affected by adjusting values C6 through C8.

The Leksell GammaPlan [LGP (Elekta)] treatment planning system calculates the dose at each point by summing the contributions from the 201 radiation beams, where exponential attenuation $\left(\mu =0.0063\;{\text{cm}}^{-1}\right)$ is used to account for the tissue thickness traversed.^(2)^ The shape of the patient's head is modeled based on 24 skull points found using a skull measurement sphere and a special ruler that measures the distance to the scalp (Fig. 1). Measurements are made at the top of the head and at selected points^(3)^ falling on four concentric rings labeled A, B, C, and D (Fig. 2).

**Figure 3:**
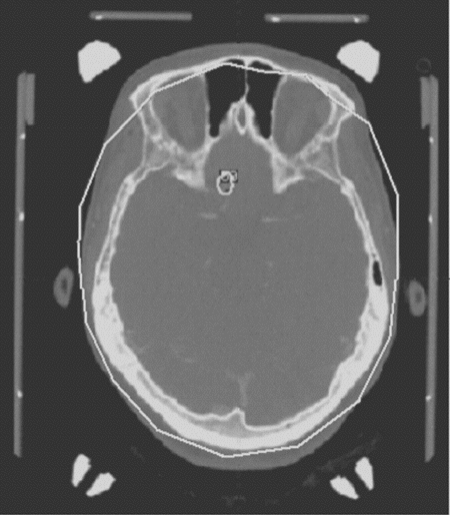
Skull model (solid white line) superimposed on a computed tomography image. Note the large discrepancy between the skull model and the actual patient contour in the anterior and posterior portions of the image.

This approach contrasts with the procedure used for external‐beam radiotherapy treatment planning, in which the patient contour is determined from computed tomography (CT) images. Gamma Knife treatment planning is generally performed using only a stack of magnetic resonance (MR) images adequate to delineate the lesion, because the skull measurement approach eliminates the need to collect a CT or an MR scan of the entire head. However, the resulting skull model can vary substantially from the actual patient contour (Fig. 3) for several reasons. First, measurements can be difficult to collect in patients with thick hair or substantial adipose tissue. Second, substantial geometric approximations can result when only 24 measurement points are used. Head contours for rotated frame placements and features such as the eye sockets, nose, and deformations because of craniotomy are not well modeled. Discrepancies between the skull model and patient contour will result in differences between the LGP and delivered doses, providing the motivation for the present research.

The work reported here investigates the effect of skull model discrepancies on treatment times for both theoretical test cases in LGP and for actual patients. The patient studies examined the effect on total treatment time for corrections to the 24‐point skull model values based on the patient images and the effect of using the actual patient contour determined from CT or MR scans.

## II. METHODS

### A. Discrepancy simulations

The impact of skull model discrepancies was first investigated by mimicking a uniform 1‐mm or 5‐mm skull radius error in LGP for a centrally located shot (x, y, z)=(100 mm, 100 mm, 100 mm) and uniform skull radius (initially 80 mm). However, in practice, skull discrepancies are seldom uniform; they generally appear as localized overestimations and underestimations. Furthermore, the magnitude of the error also depends on shot location.

We investigated the effect of localized errors for various shot positions by considering a series of treatments, each consisting of a single 8‐mm shot ranging from (x, y, z)=(100 mm, 100 mm, 100 mm) at the centre of the head to (100 mm, 30 mm, 100 mm) at the back of the head, incremented by Δy=10 mm (Fig. 3). The treatment times found with all skull measurements set to 80 mm were compared with those calculated using C7=85 mm, C6=85 mm, and C6‐to‐C8=85 mm.

### B. Manual skull measurement adjustment

At the Winnipeg Centre for Gamma Knife Surgery, CT scans of the whole head in addition to MR scans encompassing the lesion have been routinely acquired for most patients. Although the primary purpose of the scans is to check for MR image distortion, they are also useful for assessing the fit of the LGP skull model with the actual patient contour.

As part of the quality assurance performed for each treatment plan, the skull measurements are adjusted to improve agreement with the patient's CT or MR images. The total LGP treatment times associated with the measured skull shape are compared with those from the adjusted skull shape.

The cases chosen for this investigation (Table 1) are representative of those encountered in routine clinical practice: trigeminal neuralgia and acoustic neuroma treatments represent centrally located shots, and rotated frame placements such as those associated with uveal melanomas represent peripheral lesion treatments. Because the LGP skull model does not always correctly account for the narrowing of the head toward the neck, inferior lesions were included in the list. Multiple metastases are located at various positions within the head, providing representation for a variety of possible tumor locations. The squamous cell scalp treatment refers to the intracranial portion of a very advanced tumor. This rare case represents an extreme situation in which the 24‐point model was completely inadequate to describe the complex shape of the patient's head.

**Table 1:** Cases for which skull model assessment was performed

Treatment site	Cases (*n*)
Trigeminal neuralgia	5
Acoustic neuroma	5
Rotated frame placements	5
Inferior lesions	4
Multiple metastasis	4
	(37 matrices)
Squamous‐cell scalp lesion	1

### C. Skull model limitations

In some instances, the relatively coarse geometric sampling offered by only 24 measurement points led to substantial discrepancies remaining between the adjusted skull radii and the shape of the patient's head. We assessed the impact of these discrepancies for the cases listed in Table 1 by comparing the treatment times for the adjusted skull model with those calculated for the patient contour extracted from MR or CT images.

**Figure 4:**
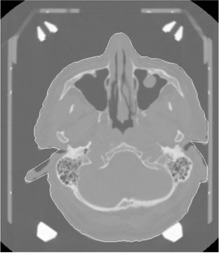
Skull contour (white line) extracted by contour program used as input for the dose verification program.

The comparison was performed using in‐house dose verification software^(4)^ that is used in routine clinical practice for validation of treatment times generated by LGP. Like LGP, the in‐house software requires entry of the 24 skull distance measurements; however, for the purpose of this research, the software was modified to accept the actual patient contour. In addition, the shot details (location, weight, helmet, γ angle, time) and maximum dose value and location are required input. The patient contour (Fig. 4) was extracted from CT or MR data using an in‐house software program written in IDL 6.0 (ITT Visual Information Solutions, Boulder, CO). This process was automated, with the option of manual corrections near features such as posts and pins. Sample points were extracted every 2 degrees, corresponding to a maximum distance of 3.5 mm between points in the plane of the CT slice, and 1.25 mm in the craniocaudal direction (that is, CT slice thickness). The 2‐degree sample spacing and ability to manually adjust the contour allows for a depth accuracy of ~1.5 mm to be achieved for 0.6×0.6×1.25‐mm voxels, because the contours are relatively smooth and do not exhibit irregularities smaller than 3.5 mm. The only exception to this statement would be in the vicinity of the ears and ear canals: the ears and ear canals were contoured as shown in Fig. 4.

## III. RESULTS

### A. Discrepancy simulations

**Figure 5:**
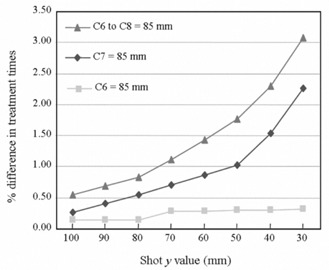
Percentage difference in treatment times for single‐shot treatments generated with all skull measurements set to 80 mm, and for the same treatments with the indicated discrepancies from 80 mm.

A uniform 1 mm or 5 mm error in all skull measurements affected treatment time for a centrally located shot by 1.5% and 3.4% respectively. The error in treatment time as a function of shot location ranged from <0.5% to about 3.0% (Fig. 5).

### B. Skull measurement validation

The percentage difference in LGP treatment times between the measured and adjusted 24‐point skull model values was calculated for 24 patient cases (Table 2).

**Table 2:** Effect of skull measurement adjustments on treatment times for the cases listed in Table 1

Treatment site	$\Delta_{\text{av}}\;(\%)$	$\Delta_{\min}\;\text{to}\;\Delta_{\max}\;(\%)$
Trigeminal neuralgia	0.2	0.0 to 0.4
Acoustic neuroma	0.3	−0.8 to 0.4
Rotated frame placements	0.2	−0.1 to 0.6
Inferior lesions	0.5	0.0 to 1.8
Multiple metastasis	0.3	−0.5 to 0.1
Squamous‐cell scalp lesion	0.5	

Av=average; min=minimum; max=maximum.

### C. Skull model limitations

The percentage difference in treatment times between the adjusted and image‐based skull contours (Table 3) was calculated using the in‐house dose verification software.^(4)^ The calculations performed by the software are similar to those performed by LGP^(2)^ and are described in detail elsewhere.^(4)^ The average and maximum deviations between point doses calculated using the in‐house verification software and using LGP were 0.2% and 3.7% respectively for 49 test cases.^(4)^ Note that the percentage differences listed in Table 3 compare results found using only the in‐house software, thereby eliminating any systematic differences between the in‐house software and LGP.

**Table 3:** Percentage difference in treatment times resulting from use of patient contours for the cases listed in Table 1

Treatment site	$\Delta_{\text{av}}\;(\%)$	$\Delta_{\min}\;\text{to}\;\Delta_{\max}\;(\%)$
Trigeminal neuralgia	0.4	0.1 to 0.9
Acoustic neuroma	0.4	−0.3 to 0.6
Rotated frame placements	1.2	−1.4 to 2.7
Inferior lesions	0.7	−0.6 to 1.3
Multiple metastasis	0.8	−2.5 to 3.3
Squamous‐cell scalp lesion	4.1	

Av=average; min=minimum; max=maximum.

**Figure 6:**
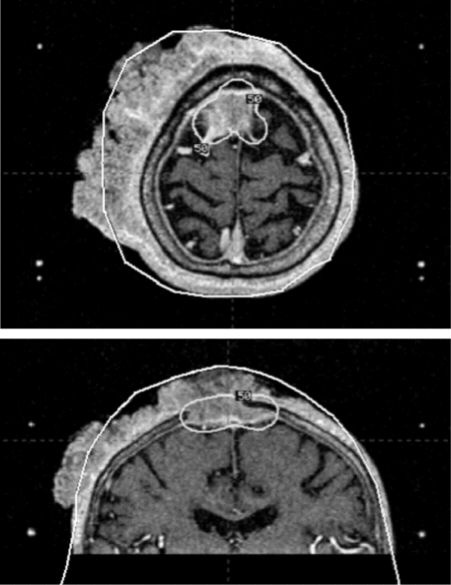
Axial (top panel) and coronal (bottom panel) view of the squamous‐cell scalp lesion, showing the 50% prescription isodose line, and of the adjusted skull model (solid white line). To avoid irradiating the skin graft, a decision was made to perform the Gamma Knife irradiation before removing the extracranial portion of the tumor.

The results for the squamous cell scalp lesion are particularly notable. Substantial disagreements remain despite considerable effort to adjust the skull model to optimize the fit with the patient contour (Fig. 6), given that a 24‐point model is not capable of dealing with such extreme variations in shape.

## IV. DISCUSSION

Fig. 5 clearly demonstrates the impact of shot position on treatment time errors resulting from skull model discrepancies. Centrally located shots are largely unaffected by overestimations (or underestimations) in the skull contour. However, as the shot moves toward the discrepancy, and as the dosimetric contribution of beams passing through the incorrect portion of the skull model increases, the error in treatment time also increases. For example, although the magnitude of the discrepancy was the same for curve C7=85 mm and C6=85 mm, the error in treatment time was smaller for curve C6=85 mm, because the shot was located farther from the skull discrepancy. A large error in the posterior (or anterior) portion of the skull model is not uncommon (Fig. 3) and is mimicked by the C6‐to‐C8=85 mm curve. Large treatment time errors can result for nearby shots in this scenario. Although the present study does not explicitly consider the effect of skull shape discrepancies on LGP isodose lines, changes in the treatment time and isodose lines would be expected to be closely correlated.

Adjusting the skull measurements to improve the fit with the imaging data resulted in only a small change in treatment times. Nevertheless, we will continue to adjust skull measurements, because they are also used to assess patient clearance. In numerous instances, tight clearances were reclassified as collisions after the skull shape was adjusted.

The comparison of treatment times between the adjusted skull measurements and actual patient contour yielded larger discrepancies. For centrally located shots such as those associated with trigeminal neuralgia and acoustic neuromas, the error in treatment time was still small. That finding is not surprising considering the results of the theoretical analysis, which demonstrated that, for centrally located shots, the effects of local overestimations (and underestimations) in the skull contour tend to cancel out. However, as the shot moves closer toward the skull contour (as for the rotated frame placements), the impact of skull model discrepancies becomes much larger. On average, the LGP head model works well for inferior lesions and for metastases. However for metastases, errors greater than 3% can result, depending on the location of the lesion. A 4.1% error was found for the squamous cell scalp lesion, which combined large skull discrepancies and nearby shots.

Ideally, future versions of the LGP software should include an option to use the actual patient contour from CT or MR data. Alternatively, a larger number of model points could be incorporated.

Another factor influencing treatment times and isodose distributions is patient inhomogeneities. LGP assumes the head to have a uniform density and does not account for attenuation differences in bone and air cavities or for boundary effects at interfaces. These topics are investigated elsewhere.^(^ ^5^ ^–^ ^8^ ^)^ Data from CT imaging, which is not routinely collected at most institutions, together with a more sophisticated dose calculation algorithm, would be required to address these issues.

## V. CONCLUSIONS

Treatment times were found to be relatively insensitive to local discrepancies in the skull contour. Thus the LGP head model provides a quick and convenient approach for determining the shape of the patient's head for dose calculations. However caution must be exercised, because in some extreme cases (such as peripheral shots), discrepancies as large as ~5% can result.

## ACKNOWLEDGMENTS

Many thanks to K. Nakonechny for reviewing this manuscript.
